# Leukemia-Induced Cellular Senescence and Stemness Alterations in Mesenchymal Stem Cells Are Reversible upon Withdrawal of B-Cell Acute Lymphoblastic Leukemia Cells

**DOI:** 10.3390/ijms22158166

**Published:** 2021-07-29

**Authors:** Natalia-Del Pilar Vanegas, Paola Fernanda Ruiz-Aparicio, Gloria Inés Uribe, Adriana Linares-Ballesteros, Jean-Paul Vernot

**Affiliations:** 1 Fisiología Celular y Molecular, Facultad de Medicina, Universidad Nacional de Colombia, Bogotá D.C. 111321, Colombia; npvanegasa@unal.edu.co (N.-D.P.V.); pfruiza@unal.edu.co (P.F.R.-A.); 2 Servicio de Patología, Laboratorio de Hematología Especial y Citometría de Flujo, Fundación Hospital de la Misericordia, Bogotá D.C. 111071, Colombia; guribeb@homifundacion.org.co; 3 Oncohematología Pediátrica, Fundación Hospital de la Misericordia, Universidad Nacional de Colombia, Bogotá D.C. 111071, Colombia; talinaresb@unal.edu.co; 4 Facultad de Medicina, Instituto de Investigaciones Biomédicas, Universidad Nacional de Colombia, Bogotá D.C. 111321, Colombia

**Keywords:** B-cell acute lymphoblastic leukemia (B-ALL), mesenchymal stem cells (MSC), leukemic niche (LN), senescence, bone marrow microenvironment, reversible senescence

## Abstract

Leukemic cell growth in the bone marrow (BM) induces a very stressful condition. Mesenchymal stem cells (MSC), a key component of this BM niche, are affected in several ways with unfavorable consequences on hematopoietic stem cells favoring leukemic cells. These alterations in MSC during B-cell acute lymphoblastic leukemia (B-ALL) have not been fully studied. In this work, we have compared the modifications that occur in an in vitro leukemic niche (LN) with those observed in MSC isolated from B-ALL patients. MSC in this LN niche showed features of a senescence process, i.e., altered morphology, increased senescence-associated β-Galactosidase (SA-βGAL) activity, and upregulation of p53 and p21 (without p16 expression), cell-cycle arrest, reduced clonogenicity, and some moderated changes in stemness properties. Importantly, almost all of these features were found in MSC isolated from B-ALL patients. These alterations rendered B-ALL cells susceptible to the chemotherapeutic agent dexamethasone. The senescent process seems to be transient since when leukemic cells are removed, normal MSC morphology is re-established, SA-βGAL expression is diminished, and MSC are capable of re-entering cell cycle. In addition, few cells showed low γH2AX phosphorylation that was reduced to basal levels upon cultivation. The reversibility of the senescent process in MSC must impinge important biological and clinical significance depending on cell interactions in the bone marrow at different stages of disease progression in B-ALL.

## 1. Introduction

Diverse and complex cues from the bone marrow (BM) microenvironment are essential to achieve a functional hematopoietic stem cell (HSC) and hematopoiesis [1,2,3]. Leukemic cells growing in the BM induce a very stressful condition and important alterations in the BM microenvironment, affecting the composition, organization, and physiology of the different cell populations [4,5,6,7]. In particular, these modifications include the elaboration of an evolving BM supportive niche for leukemic cells that would be responsible for the development of chemotherapy resistance and disease relapse [8,9]. The BM niche is the main site of relapse in pediatric B-cell acute lymphoblastic leukemia (B-ALL) [10], suggesting that privileged niches are formed in this location to support leukemic cell growth [11]. Importantly, these adverse effects were reverted when leukemic cells were dissociated from the stromal niche [7,12], suggesting that adherence of leukemic cells to stroma and the different changes induced in stromal cells are not only central to their survival but also a valid target for novel therapeutic strategies [13,14,15,16]. The mesenchymal stem cells (MSC), a main component of the BM niche, are responsible in part for these supportive and protective functions [17,18]. This is achieved basically by leukemic cell attraction via the CXCL12/CXCR4 chemokine axis and strong binding through a wide assortment of cell adhesion molecules [19,20]. Secreted cytokines, chemokines, and growth factors, and other soluble components are also responsible for the observed effect in this two-way interaction [21,22].

The characterization of other changes that may occur in the MSC after persistent and dynamic interactions with the leukemic cells is essential for the development of novel therapeutic strategies. Yet, the information related to MSC alterations in the leukemic BM microenvironment is limited, except perhaps for acute myeloid leukemia (AML) and chronic myeloid leukemia (CML) [8,23,24,25]. In an in vitro leukemic niche (LN) model [26], we have recently shown that B-ALL cell lines induced in MSC a significant increase in senescence-associated β-galactosidase (SA-βGAL) activity together with an augmented p53 gene expression and cytoplasmic reactive oxygen species (ROS) production, and a subsequent cell cycle arrest [27]. Furthermore, an altered MSC secretome, due to leukemic growth and dependent on p53 gene expression, has been reported, with a major increase in the amount of the pro-inflammatory cytokines CCL2, IL-6, and IL-8 [28,29,30]. A lower cell proliferation and the induction of cellular senescence were also reported in a mouse model of T cell acute lymphoblastic leukemia (T-ALL) [31]. MSC from patients with multiple myeloma also showed cell growth arrest and senescence features [32,33,34,35], suggesting that at least some of the alterations induced by leukemic cells are similar in the different cellular contexts of leukemia subtypes. Furthermore, essential stemness features of MSC, i.e., multipotent differentiation and self-renewal capacities, were affected in different hematological malignancies. In general, a lower osteoblastic differentiation potential was observed [24,27,32,33]. Recently, in an immunocompetent BCR-ABL1+ pre-B-ALL experimental model, it was shown that the number of osteoblastic cells and collagen production was decreased in the BM microenvironment [36]. All these observations may explain the observed skeletal abnormalities in ALL patients at diagnosis [37].

Importantly, it has been found that functional alterations of MSC are related to heterogeneous clinical prognosis in hematological malignancies [23]. Although MSC heterogeneity in BM has been clearly demonstrated [38,39], it seems that differences in niche activity can be caused by distinct functional states induced by microenvironmental cues rather than by clonal heterogeneity [40]. The MSC reprogramming by leukemic cells may not take into consideration the refinement of the diversity of MSC, and at the same time, common alterations and similar mechanisms may occur during leukemic progression independently of the leukemia subtype. Therefore, it would be important to make a more complete evaluation of the functional changes occurring in MSC under leukemic stress conditions, in particular, to define if they are long-lasting and how they compromise MSC viability, cell fitness, and stem cell functions. In addition, it would be relevant to evaluate which of these alterations can be traced to MSC isolated from B-ALL patients. This information would be important to understand better the BM physiological changes observed in patients and to develop novel strategies to prevent leukemic cell growth.

## 2. Results

### 2.1. A Senescence Process Occurs in MSC in an In Vitro Leukemic Niche and in MSC Isolated from B-ALL Patients

**Figure 1:**
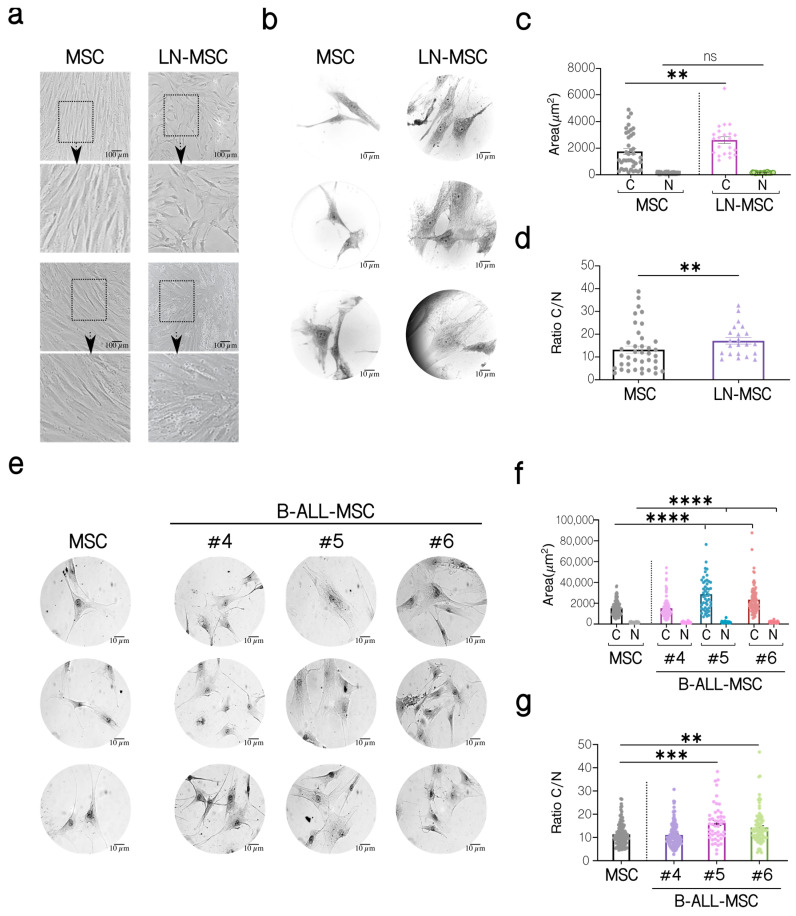
Morphological characterization of MSC co-cultivated with REH cells (LN-MSC) and MSC isolated from BM B-ALL patients (B-ALL-MSC). (**a**) Microphotographs of normal MSC and LN-MSC. Morphology was analyzed using phase contrast microscopy at 10× magnification. Scale bar, 100 µm. Bottom, a zoomed-in view from the boxed region above. (**b**,**e**) Microphotographs of a representative experiment are shown of MSC, LN-MSC, and B-ALL-MSC stained with Wright dye and acquired using an optical microscope at 100× magnification. Scale bar, 10 µm. (**c**,**f**) Determination of the cytoplasm (C) and nucleus (N) areas (µm^2^). (**d**,**g**) Evaluation of the cytoplasm to nucleus (C/N) ratio. Data are expressed as mean ± SEM. Each point on the graph represents a measurement. Two experiments with six replicates were analyzed. *p*-value ** < 0.01, *** < 0.001, **** < 0.0001 by the Kruskal–Wallis test with Dunn’s post-hoc analysis.

We have previously shown the induction of a senescent process in MSC after an in vitro co-culture for 3 days with the REH and SUP-B15 ALL cell lines [27]. To further evaluate the characteristics of MSC under leukemic growth stress, we have analyzed the cell morphology, the cytoplasm to nucleus ratio, and the immunophenotype of MSC co-cultured with REH cells (leukemic niche MSC, LN-MSC) and in primary MSC isolated from first diagnosed B-ALL patients (B-ALL-MSC). As can be seen in Figure 1, LN-MSC were larger and presented flattened morphology and granular cytoplasm (Figure 1a,b); they also showed a significant increase in the cytoplasmic area (Figure 1c) and a greater cytoplasm to nucleus ratio (Figure 1d). Likewise, except for MSC sample #4, the two other samples isolated from B-ALL patients showed the same characteristics (Figure 1e–g).

**Figure 2:**
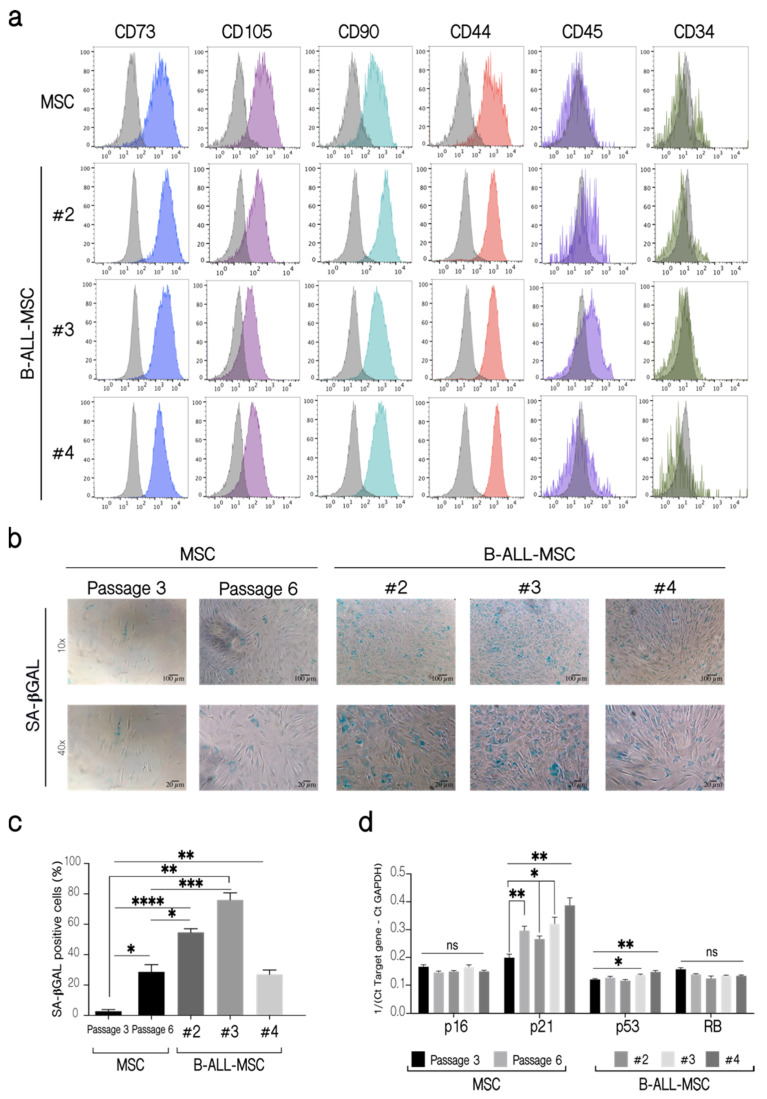
Phenotypic characterization of B-ALL-MSC and evaluation of senescence markers. (**a**) Immunophenotyping of B-ALL-MSC. Fluorescent histograms for the cell surface markers CD73, CD105, CD90, CD44, CD45, and CD34 on MSC from healthy patients and three B-ALL-MSC (#2, #3, #4). As controls, MSC from healthy patients in passages 4 (P4) were used. The grey histograms represent unlabeled cells, while color histograms correspond to the markers evaluated. The histograms shown are from a replicate. One experiment, three replicates. (**b**) SA-βGAL activity in B-ALL-MSC. As controls, MSC from healthy patients in passages 3 (P3) and 6 (P6) were used. Microphotographs at 10× (Scale bar, 100 µm) and 40× (Scale bar, 20 µm) magnification, of representative experiments are shown. (**c**) Quantification of the percentage (%) of cells positive for SA-βGAL in B-ALL-MSC. (**d**) Expression of p16, p21, p53, and RB mRNA in B-ALL-MSC. Expression of mRNA calculated from the Ct of the target gene relative to the housekeeping gene GAPDH (1/(Ct Target gene—Ct GAPDH)). Data are expressed as mean ± SEM. Four experiments with 12 replicates were analyzed. *p*-value * < 0.05, ** < 0.01, *** < 0.001, **** < 0.0001, ns (not significant) by Brown-Forsythe and Welch ANOVA test with Dunnett’s T3 post-hoc analysis.

Then we evaluated the expression of cell surface markers classically used for MSC identification and characterization [41]. We found an increase in CD73, CD90, CD105, and CD44 expression in LN-MSC compared to control MSC, while both cells were negative for CD44 and CD34 (Table S1). Except for CD105, whose expression was lower, B-ALL-MSC showed a similar pattern of cell surface expression of CD73, CD90, CD44, CD34, and CD45 compared to LN-MSC and to normal MSC (Figure 2a, Table S1).

As we found before for LN-MSC, B-ALL-MSC also showed other features associated with a senescence process. B-ALL-MSC were positive for SA-βGAL activity (Figure 2b,c) and upregulated the expression of p21 (all samples) or p53 (two samples), genes that have been associated with senescence (Figure 2d). No increase in the expression of p16 or RB was observed. As a negative control, a normal MSC from an early passage was used; a normal MSC sample in passage 6, when MSC initiate replicative senescence with the presence of some SA-βGAL positive cells (15–40%), was included. Therefore, as it occurred with LN-MSC, senescence-associated markers were also increased in B-ALL-MSC.

**Figure 3:**
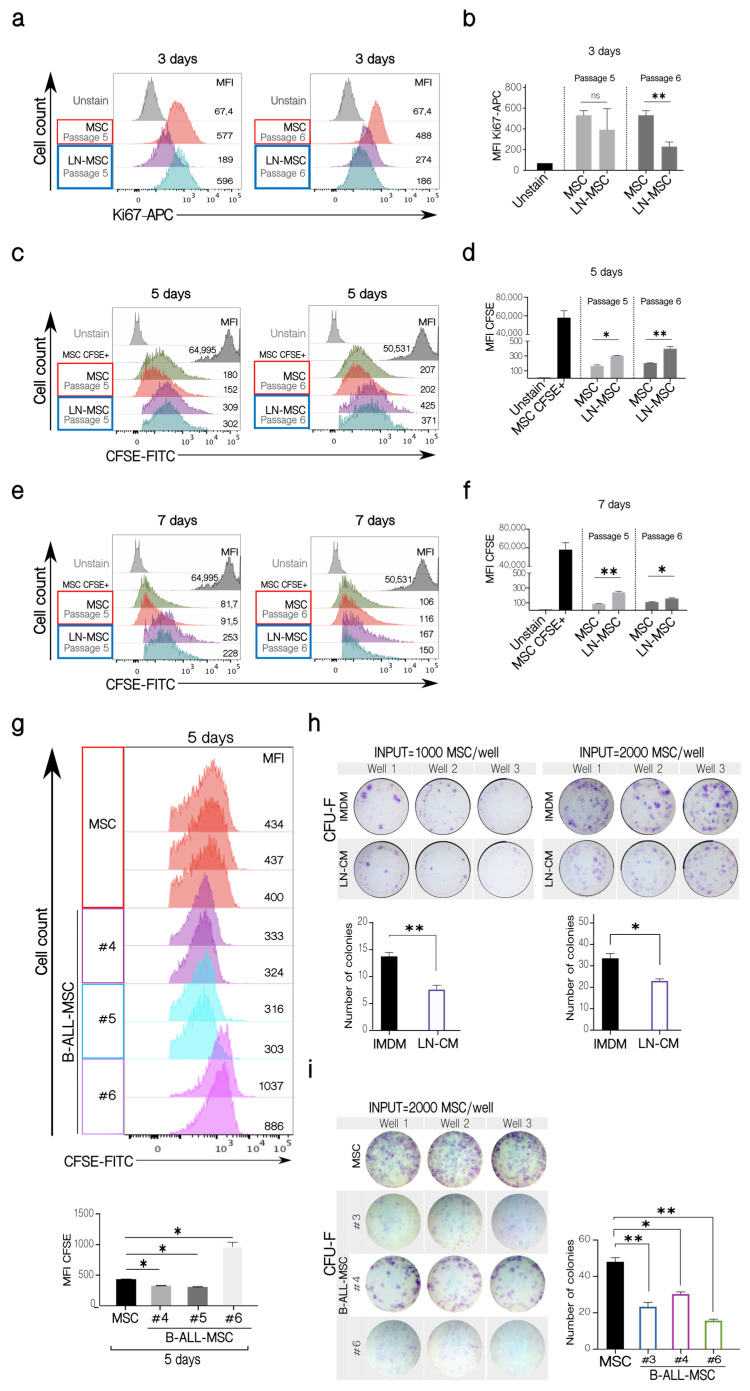
Cell proliferation and clonogenicity of LN-MSC and B-ALL-MSC. (**a**) Ki67 expression in LN-MSC. Histograms of Ki67-APC fluorescence in LN-MSC passages 5 and 6 at day 3. The histograms shown are from one experiment. FlowJo histogram overlay. (**b**) Ki67-APC mean fluorescence intensity (MFI) evaluation of LN-MSC in passages 5 and 6. Two experiments, four replicates. (**c**,**e**) Cell proliferation evaluation of LN-MSC by CFSE fluorescence. Histograms of CFSE MFI in LN-MSC passages 5 and 6 at days 5 and 7. The histograms shown are from one experiment. FlowJo histogram overlay. (**d**,**f**) CFSE MFI evaluation of LN-MSC in passages 5 and 6 at days 5 (**d**) and 7 (**f**). Two experiments, four replicates were analyzed. (**g**) Cell proliferation evaluation in B-ALL-MSC with CFSE staining. Histograms of CFSE MFI in B-ALL-MSC (#4, #5, #6) cultured for 5 days; as controls, MSC in passage 4 from healthy individuals were used; the grey histograms show the MSC freshly stained with CFSE (upper panels). The histograms (FlowJo overlay) shown are from one experiment. Lower graphs show the CFSE MFI comparison between the different samples. Two experiments with four replicates were analyzed. (**h**) CFU-F of MSC cultured with a conditioned medium obtained from a 48 h LN (CM-LN) or with IMDM supplemented with FBS. In the upper panels, photographs of the triplicate wells of two experiments seeding 1000 or 2000 MSC are shown together with the quantification of colonies in the two experiments (lower graphs). (**i**) Photographs of the triplicate wells of CFU-F of B-ALL-MSC cultured in IMDM medium with 10% FBS for 10 days (left panel). The graph on the right shows the number of colonies formed by the B-ALL-MSC. Data are expressed as mean ± SEM. *p*-value * < 0.05, ** < 0.01, ns (not significant) by Brown-Forsythe and Welch ANOVA test with Dunnett’s T3 post-hoc analysis.

To further explore the characteristics of this senescence process, MSC proliferation was evaluated. A decrease in the expression of Ki67 in the LN-MSC (Figure 3a,b, left panels) was found, and this difference was statistically significant when MSC from passage 6 were used (Figure 3a,b, right panels). To evaluate this for a longer period of time, MSC were labeled with CFSE, and the preservation of the fluorescence, indicative of less proliferating cells, was evaluated 5 and 7 days after the co-culture. As expected, lower cell proliferation was observed in LN-MSC after 5 (Figure 3c,d) and 7 days (Figure 3e,f). Similar results were obtained in MSC from passages 5 and 6 (Figure 3c–f). On the other hand, B-ALL-MSC showed variable proliferation capacity with one MSC sample showing lower and two higher cell proliferation than control cells (Figure 3g). Finally, the CFU-F assay showed less capacity to form fibroblastoid colonies in LN-MSC (Figure 3h); in this case, in order to avoid interference of the few REH cells remaining in the culture, we used a conditioned medium obtained from a co-culture of MSC and REH cells (LN-CM). Of relevance, the three B-ALL-MSC samples showed less capacity to form CFU-F (Figure 3i), despite the fact that two of the samples had previously shown slightly increased cell proliferation. Together, these experiments showed that recently isolated MSC from B-ALL patients present similar alterations in terms of morphology, cell surface markers, colony formation capacities, and, importantly, induction of a senescent process, as happens in LN-MSC.

### 2.2. Differential Regulation of MSC Stemness Features by Leukemic Cells

**Figure 4:**
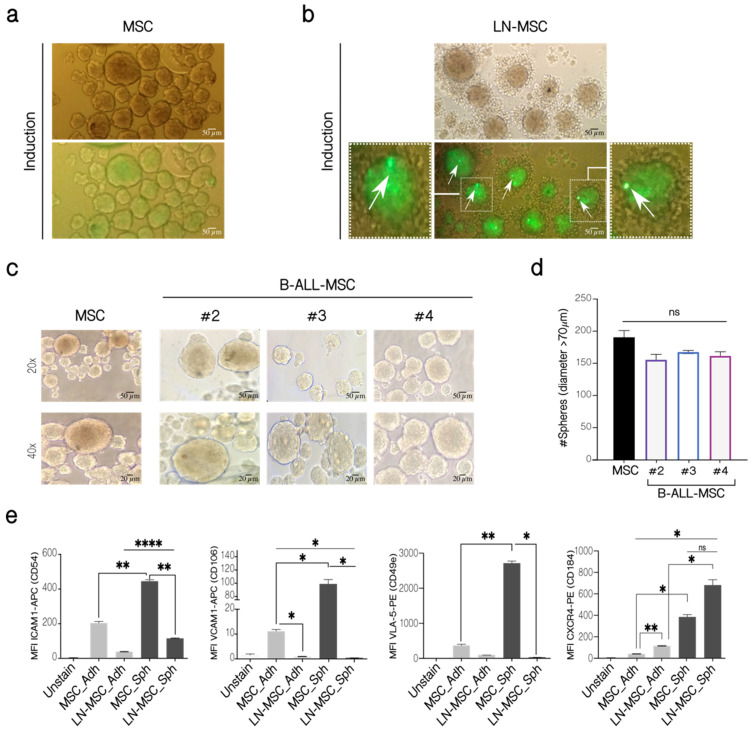
Mesensphere-forming assay of LN-MSC and B-ALL-MSC. (**a**) MSC in monoculture and (**b**) LN-MSC cultured for 5 days with sphere-inducing factors. REH cells were labeled with CFSE at the beginning of the co-cultures. Autofluorescence of MSC was observed in the FITC channel. Microphotographs at 20× under phase contrast microscopy and fluorescence microscopy of one experiment are shown. Scale bar, 50 µm. Zoomed-in views of two cells are shown on the left and right photographs with arrows indicating REH fluorescent cells. Two experiments with six replicates were analyzed. (**c**) Mesenspheres’ formation of B-ALL-MSC from three patients (#2—#4) and one healthy individual after 5 days of culture (MSC). Microphotographs at 20× (Scale bar, 50 µm) and 40× (Scale bar, 20 µm) were taken on a phase-contrast microscope of one experiment. (**d**) Quantification of the number of spheres with a diameter >70 µm. (**e**) Expression of adhesion molecules in LN-MSC mesenspheres. MFI levels for ICAM1, VLA-5 , VCAM1, and CXCR4, in adherent MSC and LN-MSC (MSC-Adh and LN-MSC-Adh), were compared to MSC and LN-MSC spheres (MSC-Sph and LN-MSC-Sph). Two experiments with six replicates were analyzed. Data are expressed as mean ± SEM. *p*-value * < 0.05, ** < 0.01, **** < 0.0001, ns (not significant) by Kruskal–Wallis test (**d**) with Dunn’s post-hoc analysis and Brown-Forsythe and Welch ANOVA test (**e**) with Dunnett’s T3 post-hoc analysis.

MSC can form clonal mesenspheres, an indication of self-renewal capacity. We have previously shown that LN-MSC induced more and larger mesenspheres than control MSC. First, we explored if these larger spheres were produced by the incorporation of REH cells within spheres by labeling REH cells with CFSE during the co-culture. MSC alone showed some autofluorescence in the FITC channel (Figure 4a, lower panel). An intense green fluorescence was observed in spheres formed by MSC in the co-cultures with CFSE-labeled REH cells, suggesting that some REH cells could be found within spheres, in spite of having washed out the majority of REH cells before the induction of mesenspheres’ formation (Figure 4b, lower panel). The exact amount of REH cells was difficult to quantify unequivocally, but between 1 and 4 intense signals of individual REH cells could be observed in each sphere (Figure 4b, arrows). This probably represents quiescent REH cells within spheres growing in a microenvironment that induces self-renewal. The proliferating REH cells, with almost complete loss of fluorescence, could be observed surrounding the mesenspheres (Figure 4b). The other fluorescence seemed to be more homogeneous and distributed throughout the sphere, suggesting that it corresponded to the autofluorescence observed in MSC. Altogether these results indicate that although some REH cells could be found inside the spheres, they neither proliferate nor contribute to the increased size observed, and that this assay reflects a real, higher MSC self-renewal capacity induced by leukemic cells. To further study this, we isolated MSC from B-ALL patients and showed that similar numbers of spheres with comparable size were formed by B-ALL-MSC when compared to control MSC (Figure 4c,d). Self-renewal seems not to be affected in B-ALL-MSC, as evaluated by this assay, and the significance of the increased self-renewal observed in our in vitro system should be further explored.

We thought that the presence of some mesenspheres with larger size induced by leukemic cells in the in vitro co-culture system could be the consequence of increased adhesion molecule expression in MSC or due to alterations of particular stemness features. First, compared to adherent MSC, MSC in mesenspheres expressed higher CD54 (ICAM-1), CD106 (VCAM-1), and CD49e (VLA-5) (Figure 4e). Nevertheless, compared to control MSC spheres, these molecules were downregulated in LN-MSC. Therefore, larger sphere formation could not be explained by upregulation of the adhesion molecules here studied, and probably represent particular features of these cells within mesenspheres. Interestingly, CD184 (CXCR4) was increased in the MSC during sphere induction and was further augmented when they were previously incubated with REH cells (Figure 4e). This would explain the increased aggregation of REH cells surrounding the mesenspheres and their higher cell proliferation observed above (Figure 4b).

**Figure 5:**
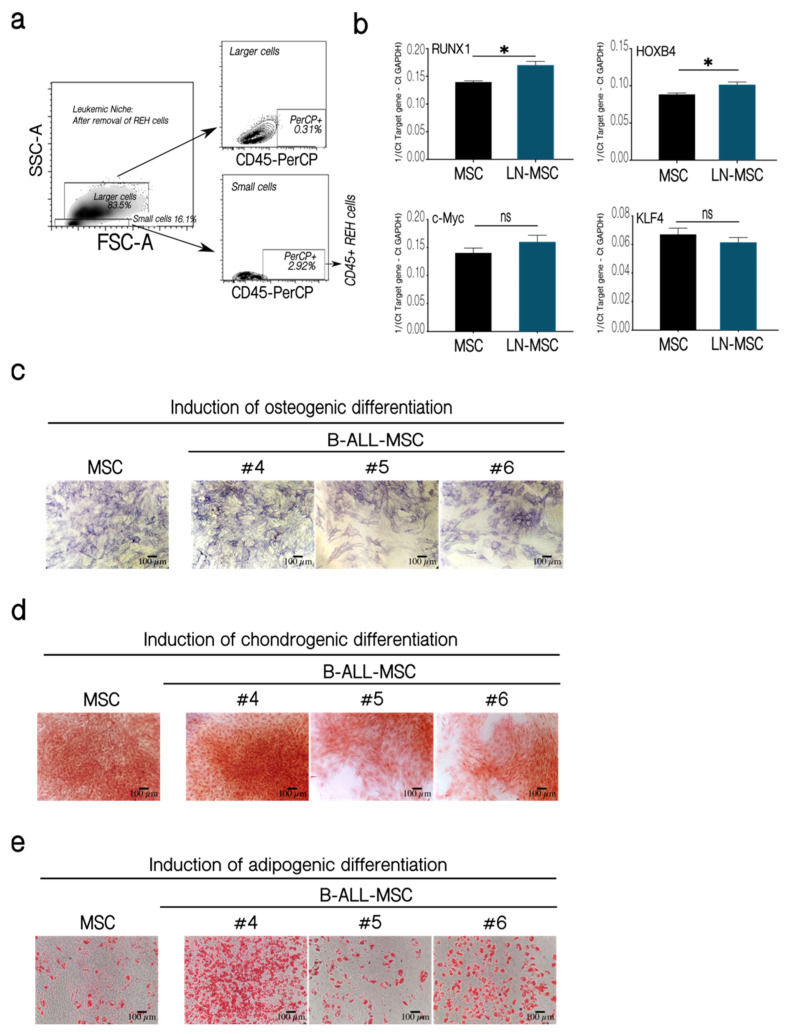
Self-renewal assessment in LN-MSC and multipotent differentiation capacity of BM-MSC from B-ALL patients. Expression of transcription factors associated with MSC self-renewal. (**a**) Less than 3% of REH cells were present in LN-MSC, as evaluated by flow cytometry. (**b**) mRNA expression of RUNX1, HOXB4, c-Myc, and KLF4 in a 3-days LN-MSC in passage 5. mRNA expression was calculated from the Ct of the target gene relative to GAPDH (1/(Ct Target gene—Ct GAPDH)). Two experiments with 10 replicates were analyzed. Data are expressed as mean ± SEM. *p*-value * < 0.05, ns (not significant) by Brown-Forsythe and Welch ANOVA test with Dunnett’s T3 post-hoc analysis. (**c**–**e**) MSC and B-ALL-MSC (#4–#6) were induced to differentiate to osteogenic and chondrogenic lineages for 21 days and to adipogenic lineage for 14 days. (**c**) Alkaline phosphatase staining for osteogenic differentiation. (**d**) Staining with Safranin O for chondrogenic differentiation. (**e**) Staining with Oil Red for identification of lipid vacuoles in adipogenic differentiation. Microphotographs at 10× (Scale bar, 100 µm) under phase-contrast microscopy of a representative experiment of MSC and B-ALL-MSC samples #4, #5, #6 is shown.

Next, we evaluated the expression of genes related to self-renewal in LN-MSC. First, we showed that REH contamination in the samples analyzed was less than 3% (Figure 5a). Both RUNX1 and HOXB4 showed a slight increase in expression compared to control MSC (Figure 5b). Although RUNX1 was also highly expressed in REH cells (not shown), the observed increase in RUNX1 was not due to the presence of this few number of REH cells. On the other hand, the expression of KLF4 and c-Myc was similar in control and MSC from the leukemic niche (Figure 5b). All these results confirm the maintenance of self-renewal function in B-ALL-MSC and a slight increase in LN-MSC.

It has been previously shown that multipotent differentiation capacity of MSC to the osteoblastic lineage is diminished in a leukemic microenvironment [24,27,32,33]. This was confirmed in two out of three B-ALL-MSC (Figure 5c, samples 5 and 6). Nevertheless, patients’ MSC showed similar (samples 5 and 6) or slightly increased (sample 4) chondrogenic (Figure 5d) and adipogenic (Figure 5e) differentiations. Altogether these results showed that MSC in a leukemic microenvironment present moderate alterations of stemness features with increased (LN-MSC) or normal (B-ALL-MSC) self-renewal properties and biased capacity to differentiate into mesenchymal lineages (in both LN-MSC and B-ALL-MSC).

### 2.3. The Chemotherapy Resistance Effect of MSC Is also Affected by Leukemic Stress

**Figure 6:**
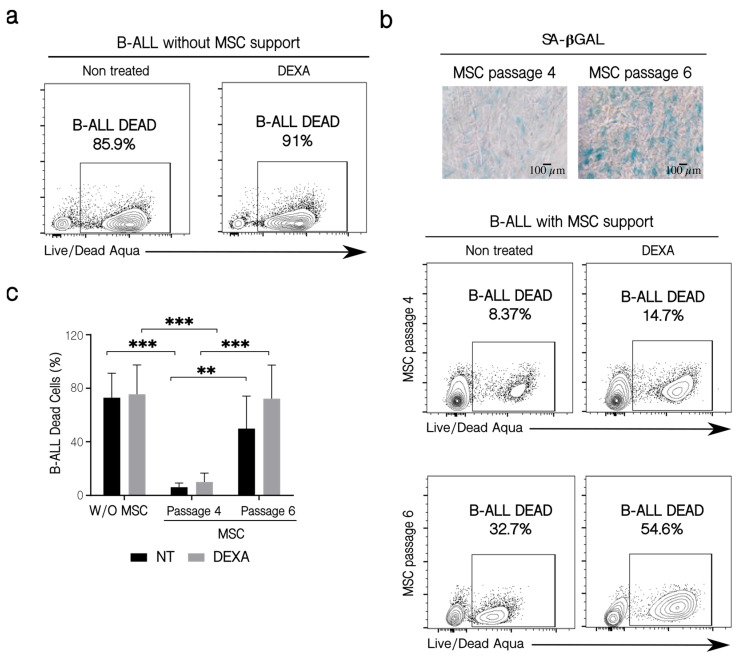
Patients’ B-ALL cell viability and their sensitization to chemotherapy. (**a**) B-ALL cell viability in the absence of MSC support. B-ALL cells were culture for 72 h alone in the presence or not of DEXA (250 nM), and then leukemic cells were collected and stained with the LIVE/DEAD Aqua reactive. Cell viability was assessed by flow cytometry. (**b**) Effect of MSC passage on leukemic cell survival in the presence of chemotherapy. Co-cultures were established for three days employing MSC from passage 4 and late passage 6, with different SA-βGAL positive cells (microphotographs 10×, Scale bar 100 µm). Before co-culturing, B-ALL cells were treated for 6 h with 250 nM of DEXA. Leukemic cell viability was determined by flow cytometry, as previously described. (**c**) Quantification of leukemic cells dead in the conditions indicated. Three experiments with six replicates were analyzed. W/O MSC: without support, NT: no treated cells. Data are expressed as mean ± SEM. *p*-value, ** < 0.01, *** < 0.001 by two-way ANOVA with Dunnett’s test post-hoc analysis.

MSC are also partially responsible for the survival and resistance of B-ALL cells to chemotherapy [12,42,43,44,45,46]. We wanted to study if the senescence process and the other changes that take place in MSC after binding of leukemic cells could affect this protective effect. For this purpose, we used B-ALL cells from patients instead of leukemic cell lines since these cells would represent a more real situation (also, B-ALL cells from patients are more dependent on mesenchymal support for survival than the leukemic cell lines). Additionally, co-cultures were established with MSC in different passages. MSC in passage 4 had very few SA-βGAL positive cells (around 5%), while cells in passage 6 had higher SA-βGAL positive cells (between 15–40%) (Figure 6b). B-ALL cells treated without support died (whether they were treated or not with dexamethasone—DEXA) (Figure 6a). In the presence of MSC, the loss of the protective effects induced by the use of an increased cell passage (and hence, a higher percentage of senescent cells) was perceived. The functional capacity of MSC to support the survival of B-ALL cells could be noticed in co-cultures even if leukemic cells were treated or not with DEXA. In the experiment shown, three times more B-ALL cells died when the mesenchymal support was provided by senescent MSC in passage 6 compared to passage 4, whether or not B-ALL cells were treated with DEXA (Figure 6b), although the chemotherapeutic agent tends to induce increase cell death. This was also true for the other two B-ALL samples tested (Figure 6c). The previous results showed that senescence alters the MSC capacity to confer support to leukemic cells, an effect that can be slightly higher in the presence of DEXA.

### 2.4. Reversal of the Early Senescence Process Induced in MSC

**Figure 7:**
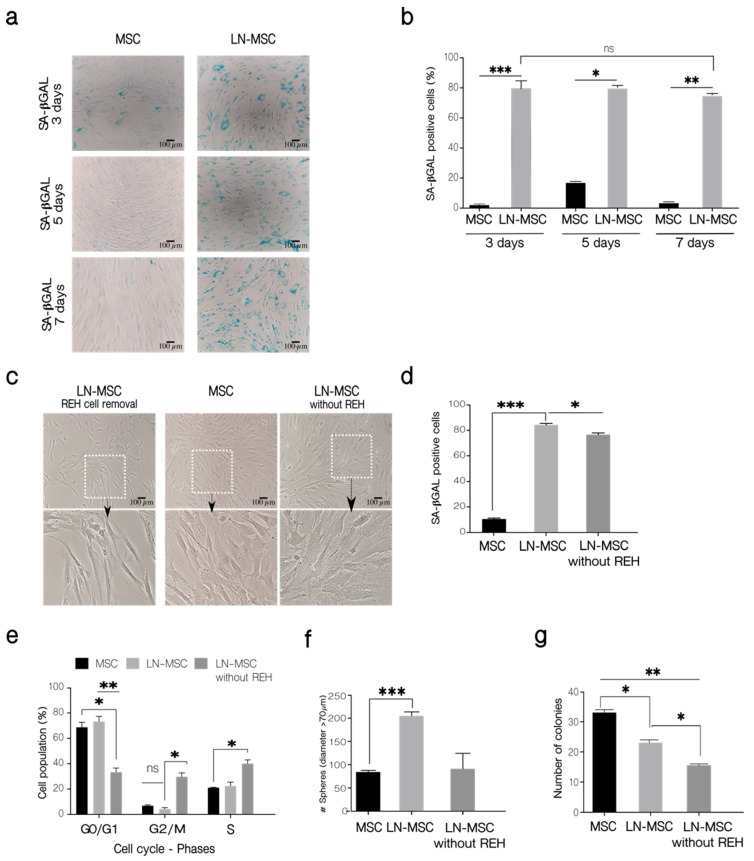
Long-term evaluation of the senescent process in MSC. (**a**) SA-βGAL activity was determined in LN-MSC at 3, 5, and 7 days. Microphotographs at 10× magnification of one replicate. Scale bar, 100 µm. (**b**) Percentage of SA-βGAL-positive cells. More than 20 cells per field, 10 fields per well or replicate. (**c**) LN-MSC morphology 3 days after washing out REH cells and culture with IMDM 10% FBS (LN-MSC without REH) of one experiment. Scale bar, 100 µm. Bottom, a zoomed-in view from the boxed region above. (**d**) SA-βGAL activity. More than 20 cells per field, 10 fields per well or replicate. One experiment with three replicates was analyzed. (**e**) Cell cycle-phases. (**f**) Number of spheres in LN-MSC (after removal of REH cells) cultured for 3 days with IMDM 10% FBS. (**g**) Number of colonies (CFU-F). In (**b**,**d**–**g**) two experiments with six replicates were analyzed. Data are expressed as mean ± SEM. *p*-value * < 0.05, ** < 0.01, *** < 0.001, ns (not significant) by the Kruskal–Wallis test (**b**,**d**,**f**) with Dunn’s post-hoc analysis and Brown-Forsythe and Welch ANOVA test (**e**,**g**) with Dunnett’s T3 post-hoc analysis.

ROS production in MSC is transient after co-cultivation with REH or SUP-B15 leukemic cell lines, restricted only to few hours (first 3–15 h for cytoplasmic ROS and first 6 or 15 h for mitochondrial ROS), after which ROS returned to basal levels [27]. This suggested to us that the leukemia-induced senescence process could also be transient. To test this, we first measured SA-βGAL activity after the establishment of prolonged LN (5 and 7 days). As can be seen in Figure 7, positivity for SA-βGAL in MSC was high (80%) after a 3-day co-culture with REH cells, but it did not vary when co-cultures were extended for 5 or 7 days (Figure 7a,b). Then we established a LN for 3 days, washout leukemic cells, further cultured the MSC for an additional 72 h and then tested MSC for SA-βGAL activity and cell cycle re-entry. MSC morphology after removing REH cells was very similar to a 6 days control culture without REH cells (Figure 7c). When SA-βGAL was detected, no diminution in SA-βGAL activity was observed after 3 days of cultivation in IMDM (not shown). Nevertheless, if the medium was replaced daily with fresh medium, a reduction in SA-βGAL activity was observed (Figure 7d). As can be seen in Figure 7e, washing out the REH cells from the co-cultures induced cell cycle re-entry of MSC, with near 40% of cells in the G0/G1 phase and the double amount in the S phase also with an important increase in the G2/M phase, compared to MSC after the initial cells 3 days in the co-cultures. Moreover, the mesensphere-forming capacity returned to normal levels when leukemic cells were removed, and MSC were washed extensively with IMDM (Figure 7f). Nevertheless, CFU-F was further reduced after removing REH cells (Figure 7g). All these results suggest that after the removal of the leukemic stress, MSC would be prone to initiate normal cell growth, and stemness functioning would be almost completely restored.

**Figure 8:**
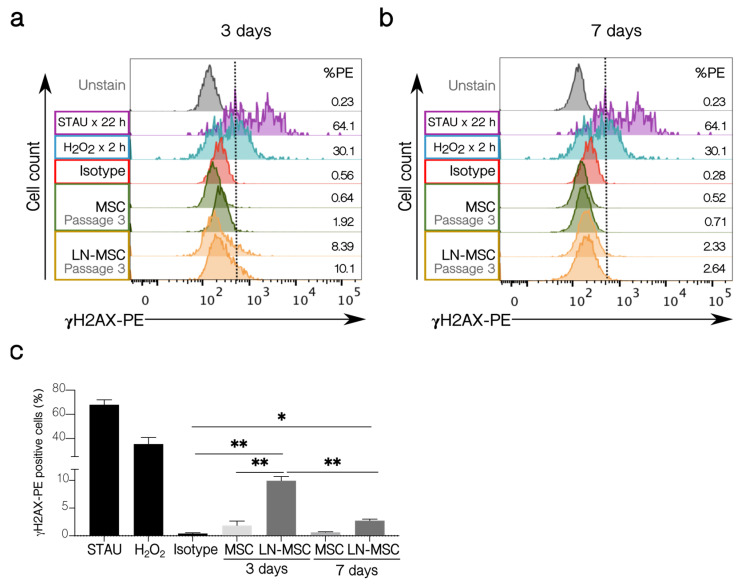
γH2AX activation in LN-MSC. Histograms with the percentage of γH2AX-PE positive cells of LN-MSC in passage 3 for (**a**) 3 days and (**b**) 7 days of co-cultures. MSC in passage 3 in monoculture were used as negative controls, and MSC in passage 3 treated with STAU at 2 µM or 5% H_2_O_2_ were considered as positive controls. The negative population for γH2AX-PE was determined based on the isotype control. %PE refers to the γH2AX positive population. The histograms shown were from one experiment. (**c**) Bar graph quantifies % γH2AX-PE positive cells. Two experiments with four replicates were analyzed. Data are expressed as mean ± SEM. *p*-value * < 0.05, ** < 0.01 by Brown-Forsythe and Welch ANOVA test with Dunnett’s T3 post-hoc analysis.

To further explore how strong the effect of leukemic cells is on MSC, we investigated if possible DNA damage could be induced by the ROS produced after interaction with leukemic cells. First, we showed that young fibroblasts treated with H_2_O_2_, or senescent fibroblasts showed increased γHA2X phosphorylation (Figure S2a,b); also, treatment with 2 µM STAU induced DNA fragmentation in fibroblast and MSC and increased γHA2X phosphorylation in fibroblast or MSC (Figure S2c,d). As can be seen in Figure 8a, only a small proportion of MSC (about 9%) showed γH2AX phosphorylation after 3 days of co-cultivation. This amount was reduced to about 3% when cultures were extended for 7 days (Figure 8b,c). Control MSC treated with STAU or H_2_O_2_ showed increased γH2AX (>60% and >30%, respectively), and these values were maintained after 7 days; untreated MSC showed no staining. This suggests that the leukemia-induced senescent process is mild and transient and that it is maintained if cells remain attached to MSC, but if the leukemic cells are removed (in vitro) or if they eventually migrate (in vivo) to other niches, then this early senescence could be halted and normal MSC proliferation and function are restored.

## 3. Discussion

In spite of their higher proliferative capabilities, leukemic cells are unable to proliferate and grow ex vivo in the absence of stromal support [11,47,48]. In the BM, leukemic cells modified the niche in their favor, severely affecting the localization and functionality of normal HSC. This is relevant, since at the same time that MSC provide support to HSC [49,50], HSC are also able to stimulate MSC proliferation and osteoblastic differentiation and inhibit senescence [51,52]. Additionally, we have shown here that, in an in vitro LN model, leukemic cells induced a senescence process in MSC, with the appearance of cells with altered morphology, reduced cell proliferation, and clonogenic capacity, and cell cycle arrest, characteristics that were accompanied by increased SA-βGAL activity and expression of p21 and p53. Importantly, the majority of these features were also observed in B-ALL-MSC and cultured for 3–4 weeks before testing. This suggests that the senescence process that we have detected in vitro has complete relevance in vivo, as it has been shown in other leukemic subtypes [31,32,33]. Accordingly, gene set enrichment analysis has shown a downregulation of genes related to the cell cycle [23], suggesting that different leukemic subtypes broadly use this mechanism to modify stromal cells.

In particular, these observed alterations could be of clinical relevance since the senescent process occurring in MSC affected the susceptibility of leukemic cells to chemotherapeutic agents. We noticed a tendency to an increased susceptibility to DEXA of B-ALL cells when the proportion of senescent MSC in culture was augmented by the use of MSC in later passages, but this deserves further experimentation or the use of more B-ALL cell samples. In this regard, it will be interesting to evaluate the consequence of senescence in the early and late stages of treatment since the chemotherapy itself may also induce senescence [53].

Of note, we observed these changes very rapidly, within 3 days of co-culture with leukemic cell lines; and as far as the leukemic cells were present, the senescent process was maintained. On the other hand, the fact that in B-ALL-MSC these changes persisted after several weeks indicates either that in vivo MSC are reprogrammed to maintain this phenotype, or alternatively, that the cues from the BM microenvironment are long-lasting during in vitro culture. This last possibility is quite reasonable for the following reasons: we have shown before that LN-MSC increased the secretion of pro-inflammatory cytokines (mainly IL-6, IL8, and CCL2) [30]; also, others and we have shown, in different cancer models, that this type of paracrine stimulation reinforces and preserves a senescent/pro-inflammatory microenvironment that promotes tumorigenesis [54,55,56,57]. Here, it is also important to mention that two gene ontology functions related to chemokine receptor binding and cytokine activity were identified in leukemia [23]. Further, in animal models, it has been shown that cytokine secretion induced long-term niche alterations [58,59].

Additional proof of this assumption came from the experiments in which we removed the leukemic stress and tested for the persistence of SA-βGAL activity. If leukemic cells were removed, and additionally the culture medium was replaced daily, a small but statistically significant reduction in SA-βGAL activity was observed. Additionally, not only normal cell morphology was almost re-established but also and importantly, MSC were able to re-enter the cell cycle and proliferate. This suggests that secreted cytokines are necessary to maintain this phenotype. Of note, the senescence-associated genes (p21 and p53) that we saw upregulated in LN-MSC and B-ALL-MSC are precisely those that are related to a reversible senescence process [60]. No increase in the irreversible senescent marker p16 was observed. This is contrary to the increased expression of p16 observed in AML, a disease that affects principally elderly patients with a natural and abundant accumulation of senescent cells in the BM [61,62].

The phenotypic analysis of LN-MSC and B-ALL-MSC showed almost equivalent expression of the cell surface markers CD44, CD73, CD90, and CD105, without the expression of CD34 and CD45, and both were very similar to normal MSC. Nevertheless, an important caveat to this reversible process was the persistent reduction in CFU-F after leukemic cells removal, in particular, since cells retaining this property are able to reconstitute both endosteal and perivascular niches [63,64].

Further evidence of mild or transient effects in B-ALL-MSC came from the evaluation of the mechanisms that could be responsible for the induction of the senescence process. Previously, we have shown that ROS production in the LN-MSC was transient [27]. We sought to investigate if this period of ROS production was sufficient to induce DNA damage and the DDR [65,66,67]. Although few cells (9%) were positive for γH2AX in the LN-MSC, this quantity was reduced to about 3%, after further co-cultivation for 4 days, suggesting that DDR response was functioning appropriately, a result that is in agreement with a transient ROS production in MSC and with the argument that stem cell populations have efficient mechanisms of repair [68,69]. In fact, the absence of genotoxic stress on MSC poses a lower risk for leukemia progression [70].

Other indications of slight modifications occurring in MSC were found when stemness properties were evaluated. We established that self-renewal was normal (B-ALL-MSC) or slightly increased (LN-MSC), as determined by the mesensphere-forming assay, with an increase in the expression of the two out of four tested self-renewal genes (RUNX1 and HOXB4 in LN-MSC). Multipotent differentiation capacity was moderately altered with reduced osteoblastic differentiation and normal or increased adipo- or chondrogenic differentiation in both LN- and B-ALL-MSC. After leukemic cell removal, self-renewal evaluation by the sphere-forming assay showed that this stemness property was preserved. Since the loss of a primitive MSC phenotype and self-renewal is linked to suppression of HSC function and leukemic chemoresistance [23], alterations in these stemness features could also be responsible in part for the increased susceptibility to chemotherapeutic agents observed above.

This presumably mild/transient effect on MSC functionality and the elucidation of a reversible senescent process taking place could be explained by the fact that we have studied LN-MSC only during the first few days of cell interaction between leukemic cells and MSC. It is possible that if leukemic stimulation is chronically maintained in vitro, more robust changes and a more defined senescent process could also have been observed. Nevertheless, these results would have eventually been biased by the fact that in in vitro cultures, replicative senescence increases after passage 7 (almost 15–17 PDT) [71], weakening the real meaning of these experiments.

On the other side, the study of the initial leukemic effect on MSC in vivo is very challenging, except for the use of experimental murine models and xenotransplantation experiments [36,72,73,74,75,76,77]. The real situation in the BM of B-ALL patients should be more dynamic than in static cultures. If it is similar to AML, in which the composition and function (perturbation of cell identity) of stromal cell clusters are affected by leukemia, then this continuous reshaping of the BM microenvironment represents an interesting and challenging hurdle [78]. Not only can B-ALL cells alter the stromal support, but also minor immune cell subpopulations may induce changes in the cell configuration of the microenvironment, in the molecular profiles related to B cell responses, and in the deregulation of physiological functions that compromise the myeloid and HSC compartments [79,80]. A detailed characterization of the complexity of the interactions in the leukemic niche will help in the future to explore new therapeutic interventions.

Moreover, MSC turnover by self-renewal or in-coming migration of new cells would eventually alleviate the situation in vivo. Furthermore, leukemic cells could move away from niches in response to stromal cues or to exhaustion of survival signals from this senescent niche, and therefore the leukemia-induced stress could be relieved temporally in MSC. When considering the evaluation of MSC isolated from patients (B-ALL-MSC), one may suppose that a previous prolonged interaction between leukemic and stromal cells would eventually induce more stable alterations in MSC. Nevertheless, it is interesting to signal that in AML, MSC alterations reflect an ongoing leukemogenic activity [23]. It was shown that patients who achieved complete remission showed similar MSC characteristics than normal MSC [23]. These results, together with our in vitro studies, suggest that instructive signals from leukemic cells and/or altered niche components depend largely on the presence of B-ALL cells or the soluble factors produced by altered MSC. In fact, in the absence of B-ALL cells, MSC reinitiated normal physiological processes. Strategies to remodel the niche toward a pro-normal/anti-leukemic niche would offer HSC opportunities to counteract the leukemic niche influence [70]. Alternatively, early therapeutic treatment would ensure that BM-MSC and other stromal cells are less affected by leukemic cell growth, guaranteeing a microenvironment prone to rapid recovery and restitution of HSC functioning.

## 4. Materials and Methods

### 4.1. MSC from Healthy Individuals and Primary MSC from B-ALL Patients

BM aspirates were collected from healthy pediatric patients (*n* = 3) undergoing orthopedic surgery without a diagnosis of hematologic disease and used for the isolation of MSC. The MNC fraction obtained from BM samples of B-ALL patients (*n* = 6) was used for the isolation of leukemic MSC. The collection of samples was performed after the signature of the informed consent by the parents following the procedure approved by the Ethics committees of the HOMI and the Faculty of Medicine, Universidad Nacional de Colombia (approved protocols 007-080-17, 11 May 2017). Ethical aspects concerning human experimentation were according to the Declaration of Helsinki.

For samples from healthy individuals, MSC were seeded in 75 cm^2^ flasks in Iscove’s Modified Dulbecco’s Medium, IMDM (with L-Glutamine, 25 mM HEPES) (GIBCO, ThermoFisher Scientific, Grand Island, NY, USA) supplemented with 10% heat-inactivated fetal bovine serum (FBS) (GIBCO, Brazil), 1% non-essential amino acids (N-EAA) (Corning, Manassas, VA, USA), and 1% sodium pyruvate (LONZA, Walkersville, MD, USA). Adherent cells were cultured until they reached 90% confluence and frozen at passage 1 in FBS with 10% DMSO solution. MSC were used in general in passages 3–5 to avoid replicative senescence. In some experiments, to have increased senescent MSC, passage 6 was used; this was signaled in the corresponding experiments. Osteo-, chondro- and adipogenic differentiation capacity (Figure S1a) and cell surface markers expression (Figure S1b) were evaluated as previously described [27]. In general, samples from the same patient were used for the same type of experiments.

For samples from B-ALL patients, MSC were seeded in the same conditions as described above, allowed to adhere to culture flasks, and after 2–3 days, the non-adherent cells were discarded. Adherent cells were cultured to 90% confluence, characterized as described above, and used immediately for some experiments or frozen. For the different experiments we arbitrarily chose 3 samples to make the comparisons.

### 4.2. Leukemic Cell Line and Primary B-ALL Cells

The ALL REH cell line was obtained from the American Type Culture Collection (ATCC; CRL-8286). REH cells were cultured in RPMI 1640 medium (GlutaMAXTM-1, 25 mM HEPES, GIBCO, ThermoFisher Scientific, Grand Island, NY, USA) supplemented with 10% FBS, 1% N-EAA, 1% sodium pyruvate, without the use of antibiotics or antifungals. The cells were maintained at 37 °C, 5% CO_2_, and a humidified atmosphere.

For the experiments related to chemotherapeutic agent sensitization, primary human BM leukemia cells from pediatric B-ALL patients (*n* = 3) were collected at diagnosis by the Paediatric Oncohematology Service of the Fundación Hospital de la Misericordia (HOMI) after the signature of the informed consent by their parents, following a procedure approved by the Ethics Committees of the HOMI and the Faculty of Medicine, Universidad Nacional de Colombia (approved protocols 007-080-17, 11 May 2017). The diagnosis was established according to WHO criteria, and blasts characterization was performed by flow cytometry (FACScanto II, Becton Dickinson Biosciences) according to the European Euroflow (www.euroflow.org, accessed on the 18 May 2020). Data were analyzed using Infinicyt Software, v. 2.0 (Cytognos SL). Mononuclear cells (MNC) were isolated by density gradient centrifugation (Ficoll-Histopaque, Sigma-Aldrich, Merck, Darmstadt, Germany) at 400× *g* for 40 min. Freshly isolated MNC were maintained in RPMI 1640 medium supplemented with 10% FBS (GIBCO, Brazil), 1% N-EAA (Corning, Manassas, VA, USA), and 1% sodium pyruvate (LONZA, Walkersville, MD, USA) or were frozen in 90% FBS and 10% DMSO and stored in liquid nitrogen until use.

### 4.3. Co-Culture of MSC with Leukemic Cells

BM-MSC from healthy donors were thawed, cultured, and used in passages 2–5 in the different experiments. Cells were trypsinized, seeded at 6–15 × 10^4^ cells/cm^2^, and cultured until they reached 80% confluence. REH cells were seeded over a MSC monolayer at a ratio of 1:3 or 1:5 (MSC:REH), depending on the experiment. The co-cultures were incubated for 3–7 days (as indicated below) at 37 °C and 5% CO_2_ in a humidified atmosphere. After the incubation time, the unattached REH cells were removed with 1× phosphate buffered saline (PBS). REH cells adhered to MSC and MSC were trypsinized. MSC and REH cells discrimination was performed by cell surface expression of CD73 in MSC or prior labeling with CFSE (CellTrace™ CFSE Cell Proliferation Kit, Invitrogen, Eugene, OR, USA) of blast cells or MSC.

For experiments with primary B-ALL cells (sensitization to dexamethasone, please see below), co-cultures were established as described for the REH cell line. Primary blasts were thawed in FBS, centrifuged at 400× *g* for 5 min, and resuspended in supplemented RPMI 1640 medium. Cell counting was performed in a Neubauer chamber, and cell viability was evaluated with Trypan blue dye exclusion. In general, the samples used showed about 90% cell viability after thawing.

### 4.4. Evaluation of the MSC Morphological Changes Induced by Leukemic Stress

For morphological evaluation, MSC were grown on sterile 16 mm diameter coverslips, and after adhesion, REH cells were added and cultured for 3 days as described above. The culture medium was discarded, and the MSC were washed several times with 1× PBS to remove the REH cells. MSC were fixed with 4% formalin and stained with Wright’s dye. Cell morphological changes were observed with an optical microscope with an objective at 100× (Axiovert 40 CFL Zeiss fluorescence microscope) and photographed (10 random images from each of the three wells per condition). Morphometric parameters were performed for each individual cell and were calculated with the area tool (given in µm^2^) using the ImageJ software v1.53a.

### 4.5. Detection of SA-β-Galactosidase (SA-βGAL) Activity

For colorimetric detection of cells positive for SA-βGAL activity, the Cellular Senescence Assay Kit (KAA002, Millipore, Merck, Darmstadt, Germany) was used. Briefly, MSC were fixed for 10 min at room temperature under darkness and washed once with 1x PBS. The labeling solution containing the substrate (X-gal) was prepared according to the manufacturer’s instructions, and the pH was adjusted to 6.0. The labeling solution was added to the cells, and the culture plate was sealed with parafilm and incubated for 4 h or ON at 37 °C in the dark. The SA-βGAL detection solution was removed, MSC were washed with 1× PBS, and ten random microphotographs per well were taken on an inverted microscope. Quantification of SA-βGAL positive cells was performed using the ImageJ software v1.53a.

### 4.6. Expression of Senescence and Self-Renewal Genes

Analyses of gene expression were performed by quantitative real-time PCR (qRT-PCR) on a 7500 real-time PCR Systems (Applied Biosystems, Foster City, CA, USA) with the PowerUp Sybr Green Master Mix (Applied Biosystems, Foster City, CA, USA). Briefly, 1000 ng RNA from each sample was reverse transcribed using the HighCapacity Kit (Applied Biosystems, Foster City, CA, USA). Primers for p16, p21, p53, RB, RUNX1, HOXB4, c-Myc, and KLF4 were synthesized by Invitrogen (Table S2). All reactions were run in triplicate, and the average values were used for quantification. Gene expression levels were normalized to the levels of the reference gene GAPDH and calculated using the inverse of the normalized expression (1/(Ct Target gene—Ct GAPDH)). The contamination or remnant of REH cells in the co-cultures was assessed by flow cytometry to evaluate possible amplifications of leukemic cells (Figure 5a).

### 4.7. Cell Surface Expression of Adhesion Molecules

For the assessment of cell surface expression of adhesion molecules, MSC were resuspended in 1× PBS and incubated for 30 min at RT in the dark with monoclonal antibody anti-human ICAM1 (CD54; (clone REA266, Miltenyi Biotec, Auburn, CA, USA)) or VLA-5 (CD49e, (clone IIA1, BD Pharmingen, San Jose, CA, USA)) or CD106 (VCAM1, clone REA269, Miltenyi Biotec, Auburn, CA, USA)) or CXCR4 (CD184, (clone 12G5, Miltenyi Biotec, Auburn, CA, USA)). Cells were washed once with 1× PBS prior to flow cytometry. Acquisition of at least 10,000 events was performed on a FACS Aria III cytometer (BD Biosciences, Franklin Lakes, NJ, USA). Data analysis was performed with FlowJo software v10.7.1.

### 4.8. Cell Proliferation Assays of MSC after Co-Culturing with Leukemic Cells

For intranuclear Ki67 detection, MSC were fixed for 20 min at 4 °C in 4% paraformaldehyde (BioLegend, San Diego, CA, USA) followed by the addition of Intracellular Staining Permeabilization Wash Buffer (1×) (BioLegend, San Diego, CA, USA) and centrifugation at 400× *g* for 7 min. The supernatant was discarded, and the cell pellet was resuspended in Intracellular Staining Permeabilization Wash Buffer (1×) and incubated with monoclonal antibody anti-human Ki67 (clone Ki67, BioLegend, San Diego, CA, USA) for 30 min at RT in the dark. Next, the cells were centrifuged at 400× *g* for 7 min, and the supernatant was discarded. Finally, the cells were resuspended in 1× PBS–0.2% BSA for subsequent evaluation in the flow cytometer.

Cell proliferation was also assessed by Carboxyfluorescein Diacetate Succinimidyl Ester (CFSE) staining. Cells were resuspended in 1× PBS with 0.1% BSA (GIBCO-Invitrogen, Grand Island, NY, USA) and incubated with 5 μM CFSE (CellTrace™ CFSE Cell Proliferation Kit, Invitrogen, Eugene, OR, USA) for 7 min at 37 °C. CFSE-stained cells were resuspended in supplemented IMDM medium and were placed on ice for 5 min and then washed three times at 400× *g* for 7 min at 20 °C with a supplemented medium. Finally, the cells were resuspended in supplemented medium and incubated for 30 min at 37 °C. As a control for CFSE staining, we evaluated the mean fluorescence intensity (MFI) by flow cytometry of freshly CFSE-stained cells.

### 4.9. DNA Fragmentation Assay

Fibroblasts were treated with staurosporine (STAU) at 2 µM for 24 h to determine DNA fragmentation. As a control, untreated cells were used. Cells were collected and centrifuged to obtain the cell pellet, which was frozen at −70 °C for subsequent DNA extraction by DNeasy Mini spin column according to the manufacturer’s protocol DNEASY BLOOD & TISSUE Kit (Qiagen, Germantown, MD, USA). Microcentrifuge tubes containing DNA for each treatment were obtained to evaluate DNA concentration by spectrophotometry (NanoDrop 2000C, ThermoFisher Scientific, Grand Island, NY) and stored at −70 °C until use. Agarose gel electrophoresis was used for qualitative analysis of DNA fragmentation. A 1% agarose gel was prepared in 1× TAE (Tris-Acetate-EDTA) buffer containing ethidium bromide. A mixture of 3 µL of DNA sample and 2 µL of loading dye (Acridine Orange) was loaded per well. The gel was run at 100 V for 50 min and visualized by ultraviolet transillumination (GeneGenius Gel Imagen System, Syngene, Frederick, MD, USA).

### 4.10. Evaluation of DNA Damage in MSC and FB

First, young fibroblasts treated with 5% H_2_O_2_ for 2 h, untreated senescent fibroblasts, and MSC in passage 3 treated with 1 µM or 2 µM STAU for 24 h were evaluated for γH2AX phosphorylation. Then, MSC and LN-MSC from the co-cultures (3 and 7 days) and positive controls of MSC treated with 2 µM STAU for 22 h and 5% H_2_O_2_ for 2 h were assessed by flow cytometry for the detection of γH2AX phosphorylation. For the determination of γH2AX (Ser139) phosphorylation, MSC were fixed with cold 1% formaldehyde and incubated at 4 °C for 15 min. Then, cells were centrifuged at 400× *g* for 5 min, and the supernatant was discarded. Cold 70% ethanol was added to the cell pellet and incubated at −20 °C for 1 h, followed by centrifugation at 400× *g* for 5 min. Cells were stained with monoclonal antibody anti-human H2A.X Phospho (Ser139) (Clone 2F3, BioLegend, San Diego, CA, USA) or mouse IgG1 isotype at a final concentration of 0.6 µg/mL in blocking buffer (1% BSA–0.2% Triton X-100 in 1× PBS) and incubated at 4 °C for 1 h in the dark. Finally, the cells were centrifuged at 400× *g* for 5 min, the supernatant discarded, and the cells resuspended in 1% BSA–1× PBS suspension buffer. Data were acquired on a FACS Aria III (BD Biosciences, Franklin Lakes, NJ, USA). Data analysis was performed with FlowJo software v10.7.1.

### 4.11. Cell Cycle Analysis of MSC

Cells were fixed with 4% paraformaldehyde for 20 min at 4 °C, washed once with 1× PBS, and permeabilized with Intracellular Staining Permeabilization Wash Buffer (1×). Cells were centrifuged at 400× *g* for 7 min, and the supernatant was discarded. The cell pellet was resuspended in Intracellular Staining Permeabilization Wash Buffer (1×) and incubated with Hoechst 33342 (2′-[4-ethoxyphenyl]-5-[4-methyl-1-piperazinyl]-2,5′-bi-1H-benzimidazole tri-hydrochloride trihydrate) (Invitrogen, ThermoFisher Scientific, Grand Island, NY, USA) (2n DNA content, G0/G1; >2n DNA content, S-G2-M) for 40 min at 37 °C in the dark. Cells were then washed with 1× PBS and acquired on a FACS Aria III (BD Biosciences, Franklin Lakes, NJ, USA). Data analysis was performed with FlowJo software v10.7.1.

### 4.12. Fibroblast Colony Forming Units (CFU-F) Assay in MSC

For the evaluation of the CFU-F capacity of MSC, cells were treated with conditioned medium (CM) obtained from the co-cultures. First, co-cultures were established as indicated above and then incubated for 48 h at 37 °C and 5% CO_2_ in a humidified atmosphere. The supernatant was collected in a 15 mL tube and centrifuged at 700× *g* for 5 min and filtered through a 0.22 µm mesh. Second, in 24-well plates, 6 × 10^4^ MSC/well in 1 mL of supplemented IMDM were seeded and allowed to adhere. The culture medium was discarded, and the CM or supplemented IMDM were added to the MSC and incubated for 72 h at 37 °C and 5% CO_2_. Re-feeding with 500 µL of fresh CM was performed at 36 h. At the end of incubation, the CM was removed, and the MSC were washed with 1× PBS. MSC were trypsinized and counted in Neubauer chamber to be seeded in 6-well plates at a density of 1000 or 2000 MSC/well in IMDM medium with 10% FBS. The new culture was incubated for 14 days at 37 °C and 5% CO_2_. The culture medium was changed every 3 days, and MSC were observed under the microscope. At the end of the incubation period, the culture medium was removed, and the MSC CFU-F were stained with 0.5% (*v*/*v*) crystal violet for 20 min at RT. The wells were washed several times with deionized water. Finally, microscopically visible colonies were counted in each of the conditions. A photographic record of each well was taken.

### 4.13. Mesensphere Formation Assay

Prior to the mesensphere formation assay, MSC or REH cells were stained with CFSE, as previously described. After the co-cultures, REH cells were removed by four washes with 1× PBS and another wash with 1× PBS + EDTA. MSC were trypsinized, centrifuged at 400× *g* for 5 min, counted, and plated at low density (15,000 MSC/well) in ultralow-adherent plates (Stem Cell Technologies, Cambridge, MA, USA). Sphere-inducing medium was prepared, containing 2% B27 supplements (GIBCO, ThermoFisher Scientific, Grand Island, NY, USA), 20 ng/mL recombinant human basic fibroblast growth factor (GIBCO, ThermoFisher Scientific, Grand Island, NY, USA), 20 ng/mL recombinant human epidermal growth factor (GIBCO, ThermoFisher Scientific, Grand Island, NY, USA), 2% insulin-transferrin-selenium (Sigma-Aldrich, Merck, Darmstadt, Germany), 0.5 µg/mL hydrocortisone (Sigma-Aldrich, Merck, Darmstadt, Germany), and 1% methylcellulose (Sigma-Aldrich, Merck, Darmstadt, Germany) in Dulbecco’s modified Eagle’s medium (DMEM)/F12 (1:1)/human endothelial (1:2) serum-free medium (GIBCO, ThermoFisher Scientific, Grand Island, NY, USA). Cultures were maintained at 37 °C and 5% CO_2_ for 5 days, and manipulation was reduced to prevent aggregation. Re-feeding of the sphere-inducing medium was performed every 48 h. Spheres were measured (diameter in µm), counted, and photographed. Images were acquired using an inverted microscope (Eclipse Model TS-100, Nikon) and an Axiovert 40 CFL Zeiss fluorescence microscope. For the induction of spheres from recently MSC isolated from B-ALL patients, the above-described protocol was followed.

### 4.14. B-ALL Cell Survival and Their Sensitivity to Chemotherapy

B-ALL cells were treated with 250 nM of DEXA (Sigma-Aldrich, Darmstadt, Germany) for 6 h in RPMI-1640 medium (GIBCO, ThermoFisher Scientific, Grand Island, NY, USA) supplemented with 1% of FBS (GIBCO, Brazil). Then, the chemotherapeutic agent was removed by washing, and leukemic cells were seeded alone or in the presence of a MSC monolayer at 70% confluence in RPMI-1640 medium (GIBCO, ThermoFisher Scientific, Grand Island, NY, USA) with 10% of FBS (GIBCO, Brazil), 1% N-EAA (Corning, Manassas, VA, USA, and sodium pyruvate (LONZA, Walkersville, MD, USA). MSC in passages 4 and 6 were employed for the evaluation of the support capacity of MSC. After 72 h, leukemic cells in monoculture were collected, and the co-cultures were trypsinized. Cells were double-stained with APC-H7- mouse anti-human CD19 (clone SJ25C1, BD Pharmingen, San Jose, CA, USA) and the reactive LIVE/DEAD Fixable Aqua (Molecular Probes, Eugene, OR, USA) for assessing cell viability. Data acquisition was done using a flow cytometer FACSAria III (BD Biosciences, Franklin Lakes, NJ, USA), and the analysis was performed employing the FlowJo software v10.7.1.

### 4.15. Long-Term Evaluations of the Senescent Process

The co-culture established between MSC and the REH cell line was used for long-term assessments of the senescent process. Briefly, the co-culture was established as described. After three days of co-culture, the leukemic REH cells were removed from the MSC by four washes with 1× PBS and washed with 1x PBS+ EDTA. The co-cultured MSC (LN-MSC), with almost no REH cells, were cultured with IMDM medium supplemented with 10% FBS (GIBCO, Brazil), 1% N-EAA (Corning, Manassas, VA, USA), 1% sodium pyruvate (LONZA, Walkersville, MD, USA) for three days. IMDM medium was withdrawn every 48 h to achieve the removal of proliferating REH cells and prevent increased cell-to-cell contact with MSC. At the end of the three-day incubation, SA-βGAL activity was assessed in the MSC, or the MSC were used for the functional experiments described above (cell cycle, CFU-F, and mesensphere-formation assays).

### 4.16. Statistics

All data are presented as the mean ± standard deviation. Nonparametric test (Kruskal–Wallis test followed by Dunn’s post-hoc test), Brown-Forsythe and Welch ANOVA test followed by Dunnett T3 post-hoc test and two-way ANOVA test followed by Dunnett’s post-hoc test were used to compare test groups. A Family-wise alpha threshold of 0.05 with 95% confidence interval to 1.96 SD was used. *p* < 0.05 was considered statistically significant. We generated statistics with GraphPad Prism9 v9.1.0 software (GraphPad).

## Data Availability

Not applicable.

## Supplementary Materials

The following are available online at https://www.mdpi.com/article/10.3390/ijms22158166/s1.
