# Supplementary material for: Leukemia-Induced Cellular Senescence and Stemness Alterations in Mesenchymal Stem Cells Are Reversible upon Withdrawal of B-Cell Acute Lymphoblastic Leukemia Cells
Source: Int J Mol Sci. 2021 Jul 29;22(15):8166. doi: 10.3390/ijms22158166 (PMC8348535; doi:10.3390/ijms22158166)
Supplement: Supplementary file 1 [file ijms-22-08166-s001.zip › ijms-1269256-supplementary.pdf]

Supplementary Materials

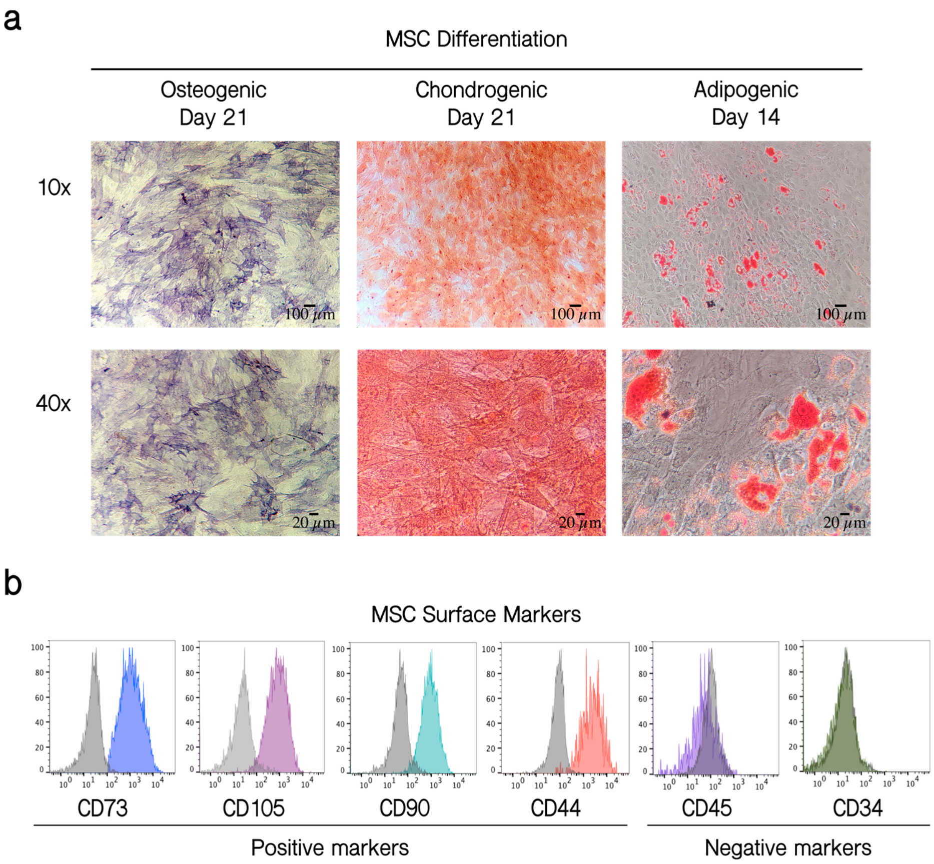

Figure S1. Characterization of MSC from healthy patients. **(a)** Differentiation potential of MSC to mesodermal lineage (osteoblasts, chondrocytes and adipocytes). Microphotographs at 10× (Scale bar 100 µm) and 40x, (Scale bar 20 µm). (**b)** Fluorescence histograms for positive (CD73, CD105, CD90, CD44) and negative (CD45, CD34) cell surface markers of MSC. Flow cytometry analysis by FlowJo software. Representative images of an experiment are shown.

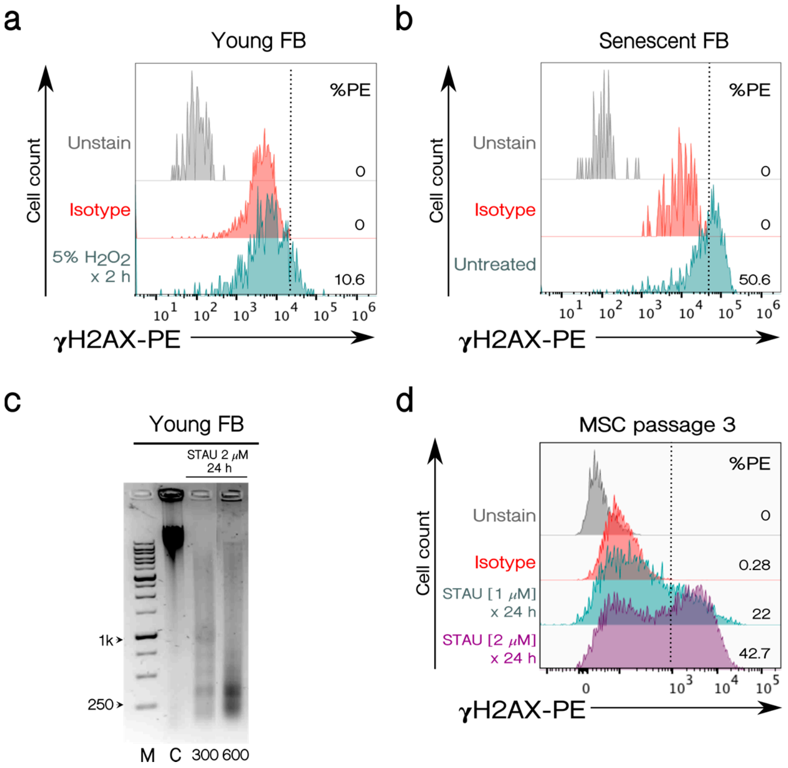

Figure S2. DNA damage in fibroblasts (FB) and MSC. (**a)** Percentage of γH2AX-PE positive cells in H_2O_2-treated young FB compared to isotype control. (**b)** Percentage of γH2AX-PE positive cells in untreated senescent FB. **(c)** Agarose gel of DNA FB treated with STAU at 2 µM for 24 h. M, molecular weight markers; C, control of untreated cells; 300 ng or 600 ng of DNA. (**d)** Percentage of γH2AX-PE positive cells in MSC treated with 1 or 2 µM STAU for 24 h.

Table S1. Cell surface marker expression in LN-MSC and B-ALL-MSC.

Cell type	*MFI Surface marker*					
	**CD73**	**CD105**	**CD90**	**CD44**	**CD45**	**CD34**
MSC	1156	828	649	617	138	484
LN-MSC	2940	2727	3007	893	120	404
**Ratio LN-MSC/MSC**	**2.54**	**3.29**	**4.63**	**1.45**	**0.87**	**0.83**
MSC	1626	379	459	1043	0.62	0.62
B-ALL-MSC #2	3910	44.8	1946	1096	0.42	0.47
B-ALL-MSC #3	2879	28.6	833	1020	1.98	0.69
B-ALL-MSC #4	2089	106	1029	2127	0.50	0.34
**Ratio B-ALL-MSC #2/MSC**	**2.41**	**0.12**	**4.24**	**1.05**	**0.68**	**0.76**
**Ratio B-ALL-MSC #3/MSC**	**1.77**	**0.08**	**1.81**	**0.98**	**3.19**	**1.11**
**Ratio B-ALL-MSC #4/MSC**	**1.28**	**0.28**	**2.24**	**2.04**	**0.81**	**0.55**

Table S2. List of primers used for RT-qPCR.

TARGET GENE	OLIGONUCLEOTIDE SEQUENCES (5′-3′)
p16	Forward, 5′-GAAGGTCCCTCAGACATCCCC-3′;<br>Reverse, 5′-CCCTGTAGGACCTTCGGTGAC-3′;
p21	Forward, 5′-GCCTGGACTGTTTTCTCTCG-3′;<br>Reverse, 5′-ATTCAGCATTGTGGGAGGAG-3′;
p53	Forward, 5′-CTGCCCTCAACAAGATGTTTTG-3′;<br>Reverse, 5′-CTATCTGAGCAGCGCTCATGG-3′;
RB	Forward, 5′-AGGATCAGATGAAGCAGATGG-3′<br>Reverse, 5′-TGCATTCGTGTTCGAGTAGAAG-3′;
RUNX1	Forward, 5′-CTGTGTAGGGGAGCCACATT-3′;<br>Reverse, 5′-CTTGTCTCCACTGAGGCACA-3′;
HOXB4	Forward, 5′-TACCCCTGGATGCGCAAA-3′;<br>Reverse, 5′-CAGGTAGCGGTTGTAGTGAAATTC-3′;
c-Myc	Forward, 5'-GAGCTGTTTGAAGGCTGGATTT-3';<br>Reverse, 5'-TCCTGTGGTGAAGTTCACGTT-3';
Klf4	Forward, 5′-TATGACCCACACTGCCAGAA-3′;<br>Reverse, 5′-TGGGAACTTGACCATGATTG-3′;
GAPDH	Forward, 5′-GACCCCTTCATTGACCTCAAC-3′;<br>Reverse, 5′-CTTCTCCATGGTGGTGAAGA-3′;
